# Factors influencing the decision to participate in medical premarital examinations in Hubei Province, Mid-China

**DOI:** 10.1186/1471-2458-13-217

**Published:** 2013-03-11

**Authors:** Peigang Wang, Xiao Wang, Min Fang, Tyler J Vander Weele

**Affiliations:** 1 School of public health, Wuhan University, Wuhan, 430071, China; 2 School of Basic Medical Science, Wuhan University, Wuhan, 430071, China; 3 Harvard School of Public Health, Boston, MA, 02115, USA

**Keywords:** Premarital couples, Premarital screening program, Influence factor

## Abstract

**Background:**

To investigate the attitudes of premarital couples towards the premarital screening program after the abolition of compulsory screening in China and to study the factors influencing participation.

**Methods:**

Between July 1^st^ 2010 to August 31st 2010, 650 people who registered for marriage at the civil affairs bureau of Wuhan, Suizhou, Zaoyang in Hubei province were studied using questionnaires. Logistic regression was used to examine the factors influencing participation in the premarital screening program.

**Results:**

The premarital screening rate was 34.8% (95% Confidence Interval: 31.0% to 38.5%). Several demographic factors (age, residence, profession), awareness, knowledge, and attitudes towards premarital screening all had significant influence on participation in the premarital screening program.

**Conclusions:**

Promotion activities and health education to improve knowledge and attitudes to premarital screening will help increase the rate of voluntary premarital screening.

## Background

The premarital screening (PMS) program is a medical examination for couples who are about to get married, in order to prevent diseases which may affect the quality of marriage and the health of future generations and to provide premarital health guidance. PMS contains different items in different regions. In China, PMS includes the testing for serious hereditary diseases, infectious diseases, and psychiatric disorders [1]. These include, in different regions, physical tests concerning height, weight, blood pressure, development status etc.; laboratory tests including blood RT, urine RT, stool RT, some organ-specific tests; chromosome testing such like RFLP; and tests for serious infectious diseases such TORCH, HIV; and mental health assessments.

The PMS program was first carried out in China from 1986, and initially was compulsory, though not free of charge. By the year 2000, the rate of premarital screening was as high as 63.4% [2]. Although the achievement and popularity of PMS were widely recognized, the fact that the policy was compulsory was strongly criticized by many foreign civil rights activists [3]. Shortly thereafter, China promulgated the new “Marriage Registration Ordinance” (1st October 2003) into the existing Marriage Law, ending mandatory premarital screening and making it voluntary. However, with the abolition of the compulsory examination, the rate of PMS dropped sharply; the number of birth defects also increased [4-6]. According to the preliminary statistics from the Ministry of Health, the rate of attending PMS in China dropped to less than 1/10 of that in previous years [7,8]. As reported in some studies, the premarital screening rate has slowly increased with the implementation of free examination policy in recent years [9], but the overall level remains low. Many foreign countries, such as the United States, had also practiced a compulsory examination policy, which similarly gave rise to disputes [10] and was eventually repealed because of cost and ethical issues [11].

PMS has been considered beneficial for the promotion of young peoples’ health and quality of life after marriage. It is an important means to attempt to help ensure more adequate prenatal and postnatal care [12]. In addition, it has an important role in the control and prevention of major sexually transmitted diseases and serious genetic diseases, especially in regions with higher prevalence of genetic disorders and diseases [13-18]. With China’s move towards modernization, perceptions can rapidly change and a number of different social factors are likely to influence people’s perception and attitudes to PMS. The decline of PMS rate will inevitably have an effect on the control of infectious diseases [19,20]. This issue has caused widespread concern in Chinese society. Given the current situation, it is important to explore what factors influence the decision of whether premarital couples participate in premarital screening.

Some studies have already noted that certain demographic factors have an effect on whether or not premarital couples participate in premarital screening [21-24]. These factors include gender, age, ethnicity, residence, profession, education, and monthly income, but these factors only explain a limited part of variation in decisions to participate. In addition, studies have confirmed that awareness of PMS [23], knowledge of PMS [25-28] and attitudes towards PMS [29-31] have significant influence on whether or not premarital couples attend premarital screening. However, prior studies of PMS in China have only considered single factors in isolation, without simultaneously controlling for other factors. The relation between the various demographic and psychological factors are complex, and correlations with a single variable may in fact also capture correlations with other related variables if these are not simultaneously controlled for. The current paper helps fill the current gap in the literature by using logistic regression to explore the influence of several related factors controlling for other factors simultaneously. We attempt to identify the main factors that influence people’s decisions to attend the PMS program. The purpose of the study is thus to examine what factors appear to influence participation in PMS and to see if it might be possible to intervene on any factors associated with PMS.

## Methods

Questionnaires to obtain cross-sectional data were administered at survey sites at the civil affairs bureaus in Wuhan, Suizhou, and Zaoyang in Hubei province, China. The surveys were administered by a team of student from Wuhan University based on a convenience sample of those at the civil affairs bureaus. The research carried out was in compliance with the Helsinki Declaration; ethical approval was obtained by Wuhan University. Wuhan is the capital city of Hubei province. It is the largest city in Mid-China. Suizhou located in the western part of the Hubei province and is an important transportation hub and with industrial concentration. Zaoyang is located in the northwest part of the Hubei province and is considered a county level city. Interviewers obtained informed consent and responses were kept confidential. The questionnaire was administered between July 1st 2010 and August 31st 2010 to 650 people who registered for marriage at the civil affairs bureau of Wuhan, Suizhou, Zaoyang in Hubei province. For each couple registering for marriage, both individuals were invited to participate in the survey; for some couples both individuals participated; for others, only one person. Of the 650 surveyed, responses were obtained from 633 for a 97.4% response rate. About 30,000-40,000 couples register for marriage each month in the Hubei province. STATA12.0 for windows was used for data processing and analysis.

In order to facilitate analysis, based on the original questionnaire data, we re-encoded certain variables (see Table 1). Profession was recoded into four categories (common clerks, leaders, scientists and teachers, farmers) according to the characteristics and status of the professions in China. The “PMS items knowledge score” is the total score concerning the knowledge the respondents had as to what PMS items are tested in the screening. Higher scores indicate more accurate knowledge of PMS. The questions include common items taken in PMS, and the participants are questioned, “how sure are you that this item belongs to PMS”, with “very sure” being set to 5 while “don’t know” is set to 1; the sum stands for the level of knowledge of the PMS items. The cronbach alpha coefficient of the variable for “PMS items knowledge score” is 0.848.

**Table 1:** The definition of variables and statistical description

**Variable**	**Sample size**	**Mean**	**Standard deviation**	**Nature/measure**	**Definition of variables**
Have you attended for the premarital screening?	630	0.35	0.477	Nominal	1=yes,0=no
Gender	632	0.44	0.497	Nominal	1=male,0=female
Age	631	27.90	5.387	Scale	Min=20, max=58
Residence	632	N/A	N/A	Nominal	0=else,1=Wuhan,2=Suizhou,3=Zaoyang
Ethnicity	632	0.97	0.162	Nominal	1=Han,0=else
Profession	633	N/A	N/A	Nominal	0=common clerks, 1=leaders, 2=scientists and teachers, 3=farmers
Education	633	3.20	1.199	Ordinal	1=junior middle school and below,2=senior middle school,3=junior college,4=college,5=graduate and above
Monthly income	633	3.08	1.107	Ordinal	1=below 800 yuan
					2=800-1500 yuan
					3=1500-2500 yuan
					4=2500-5000 yuan
					5=above 5000 yuan
Awareness of PMS	633	0.93	0.257	Nominal	1=aware,0=not aware
Awareness of the free and voluntary mode	633	0.60	0.490	Nominal	1=aware,0=not aware
Knowledge level of PMS	633	2.84	0.844	Ordinal	1=unknown, 2=know a little, 3=know generally,4=know fairly well, 5=know well
PMS items knowledge score	621	54.61	10.686	Scale	Min=14, max=70
Attitude to the necessity of PMS	630	3.65	0.644	Ordinal	1=not necessary at all,2=unnecessary,3=a little necessity,4=very necessary
Attitude to the preventive effects of PMS	631	2.54	0.533	Ordinal	1=makes no difference, 2=general effect, 3=has considerable effect

The dependent variable is an indicator of participation, evaluated by the question “Did you attend premarital screening?”. For expository purposes, independent variables were divided into four groups: (i) demographic factors (including gender, age, residence, ethnicity, profession, education, monthly income), (ii) “awareness of PMS” (including “awareness of PMS” and “awareness of the free and voluntary mode”), (iii) “knowledge of PMS” (including “knowle level of PMS” and “PMS items knowledge score”) and (iv) “attitude towards PMS” (including “attitude to the necessity of PMS” and “attitude to the preventive effects of PMS”).

Variables such as “awareness of PMS” and “knowledge level of PMS” will overlap to some extent since those who have some knowledge about it must be aware of it. However, it is not necessary that those aware of PMS know the PMS very comprehensively. The dependent variable, participation in PMS, is a binary variable, and logistic regression was used to examine associations of each variable controlling for all others.

Five multivariate logistic regression models were used to assess factors influencing the likelihood of participating in PMS. Robust standard errors were used to take into account potential clustering of responses by couple. Each model includes all of the demographic factors. The first model includes only the demographic factors; models 2–4 include the demographic factors along with just one of the three other sets of factors: model 2 includes demographic factors and the “awareness of PMS” variables; model 3 includes demographic factors and the “knowledge of PMS” variables; model 4 includes demographic factors and the “attitude towards PMS” variables; and the fifth model includes all four sets of factors. These models were chosen a priori; no variable selection methods were used.

## Results

Results show that among 633 questionnaires, 220 people participated in PMS, while 410 people had not; on 3 questionnaires the data were missing. The PMS rate was 34.8% (95% Confidence Interval: 31.0% to 38.5%). This figure is far lower than the 63.4% in 2000, but much higher than that reported by earlier studies [7,8]. Table 2 shows the results of the logistic regression analysis of the factors that influence the decision to participate. Model 1 shows that demographic factors explain only 6.9% of the total variance of the dependent variable and only some of the demographic factors have a significant correlation with the dependent variable. The variable of “age” has a significant influence. The results show that older people are more likely to attend PMS. The variable “residency” has a significant influence on the dependent variable and indicates people living in Wuhan and Suizhou are more likely to participate in PMS than those living in Zaoyang. In Suizhou, the participation rate is 49.3%, which is much higher than the general level of other regions. Profession also has some influence on participation with scientists and teachers being more likely to participate in PMS, while farmers are less likely to participate. The variables of “gender”, “ethnicity”, “education”, “monthly income” were not significantly associated with participation.

**Table 2:** Binary logistic regression model coefficients for PMS participation given different sets of covariates (B=coefficient; SE= robust standard error)

	**Model 1**		**Model 2**		**Model 3**		**Model 4**		**Model 5**	
	**B**	**SE**	**B**	**SE**	**B**	**SE**	**B**	**SE**	**B**	**SE**
Covariate										
Gender(reference group: female)	−0.111	0.207	−0.053	0.211	−0.120	0.216	−0.105	0.207	−0.040	0.219
Age	0.048***	0.018	0.039**	0.019	0.038**	0.018	0.042**	0.019	0.026	0.019
Residence in Wuhan (reference group: else)	0.670*	0.382	0.642*	0.383	0.630*	0.387	0.775*	0.391	0.706*	0.390
Residence in Suizhou (reference group: else)	1.370***	0.410	1.413***	0.415	1.214***	0.414	1.388***	0.418	1.288***	0.423
Residence in Zaoyang (reference group: else)	0.087	0.460	0.190	0.459	−0.055	0.465	0.072	0.469	0.020	0.468
Ethnicity(reference group: non-Han)	0.006	0.537	−0.271	0.520	0.054	0.538	−0.121	0.602	−0.117	0.605
Profession is leader (reference group: common clerks)	−0.115	0.213	−0.168	0.219	−0.165	0.221	−0.076	0.219	−0.135	0.228
Profession is scientist or teacher (reference group: common clerks)	0.388*	0.276	0.257	0.278	0.302	0.288	0.383	0.283	0.198	0.293
Profession is farmer (reference group: common clerks)	−0.929*	0.671	−0.737	0.663	−0.845	0.709	−0.756	0.691	−0.712	0.744
Education	−0.037	0.101	−0.091	0.104	−0.155	0.105	−0.067	0.102	−0.189*	0.107
Monthly income	−0.066	0.110	−0.042	0.111	−0.067	0.114	−0.087	0.109	−0.75	0.113
Independent variable										
Awareness of premarital screening			2.804***	1.047					2.346**	1.050
Awareness of the free and voluntary mode			0.634***	0.206					0.463**	0.217
Knowledge level of premarital screening					0.606***	0.120			0.480***	0.131
PMS items knowledge score					0.010	0.010			0.006	0.010
Attitude to the necessity of PMS							0.860***	0.191	0.766***	0.205
Attitude to the preventive effects of PMS							−0.276	0.195	−0.425**	0.206
Pearson Chi-Square	40.686***		72.967***		70.279***		62.731***		104.195***	
−2 Log Likelihood	697.500***		665.218***		656.745***		674.581***		521.946***	
Cox&Snell R-Square	0.069		0.121		0.118		0.105		0.171	
N	633		633		633		633		633	

The “awareness of PMS” variables were added to the demographic factors in the regression in model 2. The results show that, controlling for the demographic factors, those with greater awareness of PMS were, unsurprisingly, more likely to participate and also that those aware of the free voluntary mode of participation were also more likely to participate. Including the awareness variables resulted in a substantial increase in explanatory power of the model with an increase in Cox & Snell R-Square, from 6.9% to 12.1%, suggesting that this “awareness” variable group plays a central role in whether couples attended PMS or not.

The “knowledge of PMS” variables were added to the demographic factors in the regression in model 3. The variable “PMS items knowledge score” was not significantly correlated with participation. However, the variable of “knowledge level of premarital screening” has a significant influence on participation. Individuals with more knowledge of PMS were more likely participate in the screening.

The “attitude towards PMS” variables were added to the demographic factors in the regression in model 4. The variable “attitude to the preventive effects of PMS” was not significantly associated with participation. The variable “attitude to the necessity of PMS” was significantly associated.

Model 5 includes all four sets of explanatory factors. With all sets of variables the explanatory power is increased further with a Cox & Snell R-Square of 17.1%, indicating that these factors explain a moderate portion of the total variance of the dependent variable. In models 2–5, when the additional factors are added, the variable of “profession” does not remain statistically significant, suggesting that the explanatory effect of this variable is replaced by the awareness/knowledge/attitude variables. Profession may be serving as a proxy for knowledge and awareness. In model 5, when all the factors are in the model, the variable “education” becomes negatively associated: those with higher educational levels are less likely to participate; in model 1, education may have been serving as a proxy for knowledge and awareness which might have cancelled out the apparent negative association with education itself. The awareness, knowledge and attitude variables all remained statistically significant when all variables were included in the model. However, the “attitude to the preventive effects of PMS” variable became significantly and negatively associated with participation once all other variables were included in the model.

In this survey, 410 people did not participate in PMS. We conducted further investigation of the reasons for not participating in premarital screening. Each study subject who did not participate indicated the most important reason for not participating. Table 3 shows the frequency of the reasons. From the Table 3 we can see that, one of the biggest reasons leading to people not attending PMS is lack of time, accounting for 18.8% of the total number of all responses. In addition, belief that “having the usual medical checks and premarital screening is unnecessary” was also a common reason (11.5%). Nearly 8% considered PMS to simply be “going through the motions” and 7.6% believed themselves to be healthy with no need for PMS. Those who were opposed by spouse or relatives and those with fear about bad hospital service constituted only a small minority of the responses. Many did not participate in PMS for some personal reason, such as “the site of premarital screening is far away”, “fear of procedures” or “do not like hospital and other inspection sites”. Of course, some (6.1%) also did not attend for PMS due to the lack of knowledge about it, consistent with what was suggested by the logistic regression analysis.

**Table 3:** Frequency of reasons for not attend PMS

**Reasons for not attend PMS**	**Frequency**	**Percent**
Have no time	77	18.8
Site of PMS is far away	20	4.9
Do not like hospital and other inspection sites	23	5.6
Too expensive	8	2.0
Fear procedures	21	5.1
PMS is just going through the motions	32	7.8
Having usual medical check-ups and PMS is unnecessary	47	11.5
Believe self to be healthy and no need for PMS	31	7.6
Be in time for check after pregnancy	10	2.4
Opposed by spouse or relatives	1	0.2
Worry about discovering diseases and affecting relationship with spouse	5	1.2
Both believe each other	6	1.5
Afraid to encounter rogue doctors	0	0
Fear about the bad hospital services	7	1.7
PMS is no longer required for marriage registration	9	2.2
Publicity is not sufficient and lack information about PMS	25	6.1
Other reasons	49	12.0
Valid total	371	90.5
Missing	39	9.5
Total	410	100.0

## Discussions

On the basis of controlling for multiple variables, our analyses suggested that age, residence, profession, “awareness of PMS”, “knowledge of PMS” and “attitudes towards PMS” are all significantly associated with participation in PMS. Profession and education may serve as a proxy for awareness and knowledge variables and once control is made for these, education may in fact have a negative association with the decision to participate. The variables of “gender”, “ethnicity”, “education”, “monthly income” were not significantly associated with participation, consistent with some previous literature [32].

Older individuals appear to have higher rates of participation in premarital screening. This may be due to the fact that, as the age increases, people attach increased importance to health. An increasing sense of responsibility with age may also influence participation [15]. Increased concern about birth defects may also be partially responsible.

In the analyses, “residence” was significantly associated with the decision to participate.

Residents of Wuhan and Suizhou were more likely to participate than those living in Zaoyang or elsewhere. As discussed further below, the Suizhou government, in particular, places considerable importance on PMS by implementing a comprehensive examination before marriage and emphasizing free premarital screening. It gives some guidance and advice to couples before marriage and promotes PMS knowledge. The regional results suggest that local policy can exert considerable influence on PMS participation rates.

Scientists and teachers were found to be more likely to participate than those in other professions. The cultural literacy and professional characteristics of the scientists and teachers, may give them a more comprehensive and clearer understanding of PMS. Profession may in fact be serving as a proxy for knowledge and awareness of PMS and indeed our analyses which included knowledge/awareness factors in the model resulted in non-significant associations between profession and participation. Conceived of another way, knowledge and awareness may mediate the effects of profession on participation. Similar but perhaps more striking results were found for education. Education may be serve as a proxy for knowledge and awareness of PMS. Once control was made for knowledge and awareness of PMS, the education variable in fact had a negative association with participation.

After controlling the demographic factors, the awareness, knowledge and attitude variables remained significantly associated with participation. Those aware of PMS and of the free voluntary mode in the screening, those with more knowledge of PMS and those who thought PMS necessary were all more likely to participate. However, controlling for all other factors, the variable “attitude to the preventive effects of PMS" was negatively associated with participation. One possible explanation is that those who are most aware are also concerned about testing positive for various conditions. Another explanation is that those who feel positively about the preventive effects may feel they do not personally need this benefit and may have little knowledge of genetic inheritance and ignore the seriousness of hereditary disorders [25,33,34]. In fact, Chen Jinsheng, who also found a similar phenomenon in his study, pointed out that there is sometimes a high degree of “one's acts belie one’s words” between the attitudes towards PMS of Chinese people and whether one participates or not [35].

China is still in an early stage of economic development. People's living standard of living is on average still low by western standards. In some regions, medical and health care services are limited. Serious genetic diseases, infectious diseases and mental illnesses continue to be important factors that influence the people's living standards. The sharp decline of PMS rate resulted from abolishing the compulsory premarital screening policy. Some Chinese scholars have argued that the compulsory policy should be restored or a new mode of participation and screening should be considered [36,37]. Given current policy, appropriate measures should be taken to improve the people's knowledge and awareness of and attitudes towards PMS so as to increase participation rates. The results concerning the high rates in Suizhou example strongly suggests that the local government and local strategies can have a significant impact on the local residents’ attitudes to PMS. The government there established a PMS team to manage and develop strategies to promote PMS. Such strategies include providing PMS knowledge by mass media, a special fund for PMS, a counseling hotline, and providing PMS completely free of charge. Their approach suggests that governments can effectively care for and support PMS programs and provide appropriate guidance. The results of our study strongly confirm that residents’ awareness, knowledge and attitudes play an important role in the decision to participate. Resources should be utilized to intensify promotional material on the purpose and significance of PMS in a broad sustained manner. Mass media can be used in providing information about premarital screening so that residents will have a more positive attitude towards prevention. Other research indicates that educational programs can considerably improve people's knowledge of PMS [38]. Improving educational programs and including PMS in various curricula in both high school and university contexts may be important in increasing knowledge and awareness. Educational programs for PMS have proven to be effective in other settings [38].

Our study is subject to some limitations. First, the investigation sites were chosen in only three cities in Hubei province. This limits somewhat the generalizability of our findings. Second, subjective information was solicited on the questionnaires, and responses may not be fully accurate. Third, the convenience sample of the study participants obtained by surveying those who were registering for marriage at civil affairs bureaus on particular days may not be fully representative of the responses in the three cities if there are substantial seasonal or geographical variations in the patterns of those registering that are strongly correlated with the questionnaire responses. Finally, although our study gave insight into a number of important factors that influence participation and also confirmed some previous findings, while also controlling for all other variables simultaneously, the explanatory power of the model was only 17.1%. There is still a considerable portion of the variation that is not explained by the factors in this study. The decision to participate in PMS is complex and multi-faceted. Future research could attempt to explore other factors that may be associated with the decision to participate.

## Conclusions

Awareness and knowledge of premarital screening programs are associated with decisions to participate in premarital screening. Promotional activities and health education to improve knowledge and attitudes to premarital screening may help increase the rate of voluntary premarital screening.

## Competing interests

The authors declare that they have no competing interests.

## Pre-publication history

The pre-publication history for this paper can be accessed here:

http://www.biomedcentral.com/1471-2458/13/217/prepub
